# Three‐dimensional structure of the wheat β‐amylase Tri a 17, a clinically relevant food allergen

**DOI:** 10.1111/all.13696

**Published:** 2018-12-19

**Authors:** Gerhard Hofer, Sandra Wieser, Michael K. Bogdos, Pia Gattinger, Ryosuke Nakamura, Motohiro Ebisawa, Mika Mäkelä, Nikolaos Papadopoulos, Rudolf Valenta, Walter Keller

**Affiliations:** ^1^ Institute of Molecular Biosciences, BioTechMed Graz University of Graz Graz Austria; ^2^ Division of Immunopathology Department of Pathophysiology and Allergy Research Center for Pathophysiology, Infectiology and Immunology Medical University of Vienna Vienna Austria; ^3^ Division of Medicinal Safety Science National Institute of Health Sciences Kanagawa Japan; ^4^ Clinical Research Center for Allergology and Rheumatology National Hospital Organization Sagamihara National Hospital Kanagawa Japan; ^5^ Department of Allergology, Skin and Allergy Hospital Helsinki University Central Hospital Helsinki Finland; ^6^ Allergy Department 2nd Pediatric Clinic University of Athens Athens Greece; ^7^ NRC Institute of Immunology FMBA of Russia Moscow Russia; ^8^ Laboratory for Immunopathology Department of Clinical Immunology and Allergy Sechenov First Moscow State Medical University Moscow Russia

To the Editor,

Wheat is one of the most important staple foods worldwide and has been recognized as a potent food allergen source. Allergies are on the rise in western countries and the prevalence of food allergy has reached approximately 7% in children in the United States,^1^ making improved diagnostics an important goal. Not all known potential allergens of wheat have been characterized so far and the prevalence of true wheat allergy is difficult to determine due to IgE cross‐reactivity with grass pollen allergens.^2^

The wheat β‐amylase (Tri a 17) has been found to bind IgE of wheat allergic patients,^3^ but its structure and allergenic activity have not been studied. To evaluate its possible clinical relevance and to shed some light on its biochemical properties, we elucidated the three‐dimensional structure, measured the enzymatic activity, IgE‐binding capacity, and allergenic activity of the recombinant enzyme.

The β‐amylases of crop plants like wheat, soy, or barley have been under investigation for decades and the presence of two general forms has been described. One protein isoform found in all parts of the plant, termed ubiquitous β‐amylase, and one variant specific to the endosperm, featuring an elongated C‐terminal tail region are known. To date, only the ubiquitous form of wheat β‐amylase has been sequenced.^4^

We screened a wheat seed cDNA library with serum IgE antibodies from patients suffering from wheat‐induced food allergy and identified an IgE‐reactive cDNA clone which was homologous to the barley endosperm β‐amylase at its C‐terminus and to the sequence of wheat ubiquitous β‐amylase at its N‐terminus.

We then expressed a recombinant protein consisting of the ubiquitous wheat β‐amylase sequence fused with the recovered IgE‐reactive C‐terminus (named Tri a 17_clone) and the unaltered ubiquitous β‐amylase (termed Tri a 17_inactive) both as inclusion bodies in *Escherichia coli*. A refolding attempt yielded soluble, yet misfolded and aggregated protein. However, we were able to optimize the expression conditions to obtain natively folded amylase (named Tri a 17_active) (Figure S1 and S2 and the Methods section in this article's Online Repository).

The far‐UV CD spectra of the inactive proteins indicate the presence of β‐sheets and random coil signal, while Tri a 17_active exhibits the high α‐helical content of a TIM‐barrel (Figure 1B).

**Figure 1:**
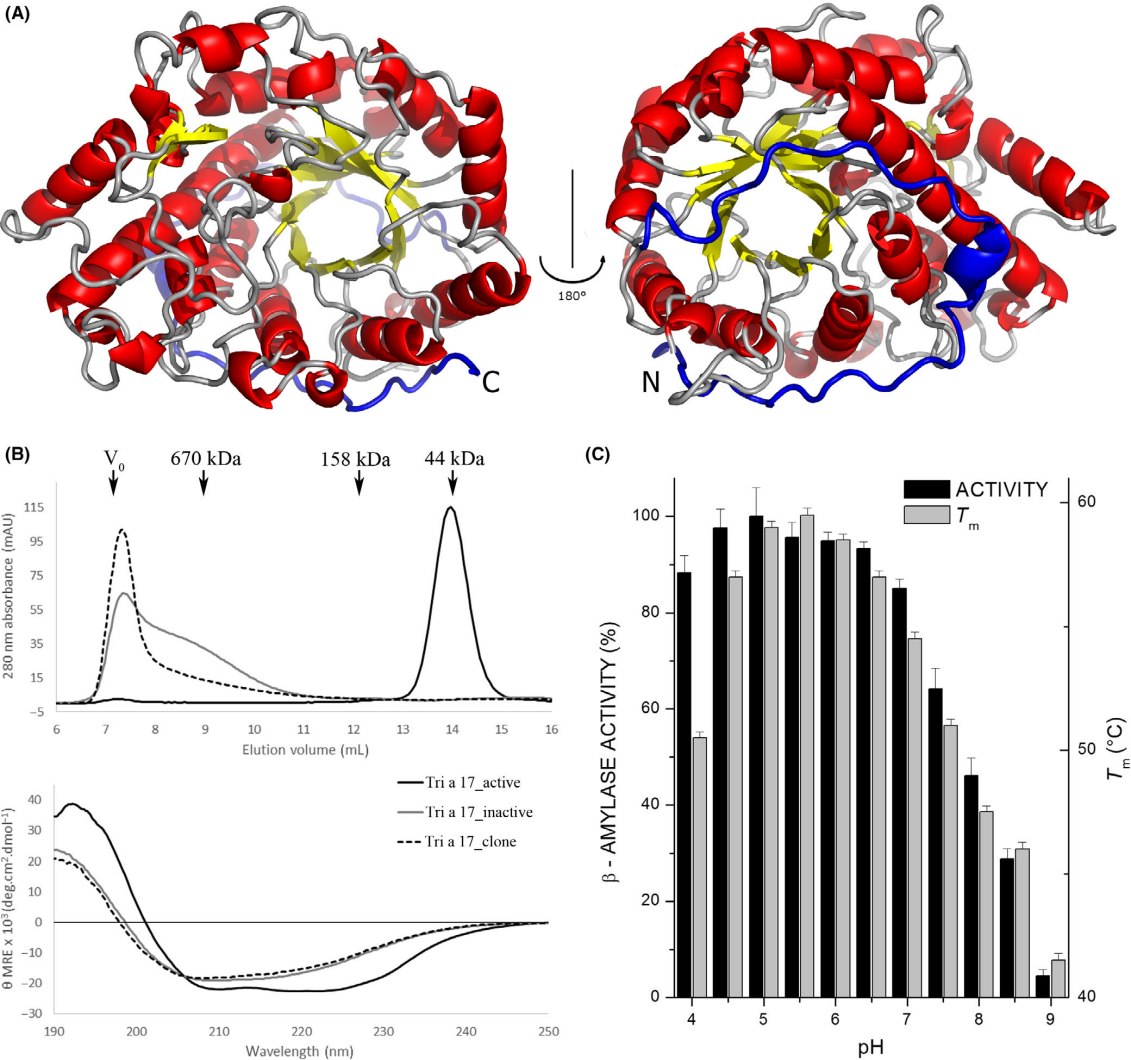
A, Cartoon model of Tri a 17 showing the (β/α)_8_‐barrel architecture with its C‐terminal portion, which corresponds to the IgE‐reactive peptide, in blue. B, Size exclusion chromatography traces of the recombinant amylases, marker protein elution volumes, and void volume are shown with arrows (top). Circular dichroism analysis of recombinant β‐amylases. The spectra are expressed as mean residue ellipticities (θ‐MRE) (*y*‐axis) at given wavelengths (*x*‐axis) (bottom). C, Percent maximum activity of Tri a 17_active as a function of pH (black). Tri a 17_active melting temperatures (T_m_) against pH as measured by DSF (gray). Data are averages of three independent measurements with error bars showing the standard deviation

Purified Tri a 17_active crystallized readily and the structure was solved by X‐ray crystallography to a resolution of 2 Å (PDB: 6GER). The structure superimposes well with the previously published structure of barley β‐amylase (rmsd = 0.62 Å when 442 of 486 Cα atoms are aligned with the PDB structure 2XFF^5^), which was used as the template for molecular replacement.

The crystal structure of Tri a 17_active shows the same (β/α)_8_‐barrel architecture found for other plant and bacterial β‐amylases (Figure 1A).

Figure 1C shows the variation in the relative enzymatic activity of the β‐amylase with respect to pH. The enzyme shows a tolerance for low pH, showing maximum activity at pH 5 and retaining over 80% of its maximum activity even at pH 4. However, at higher pH values, a marked decrease in activity is seen, with more than 50% activity lost at pH 8. In general, Tri a 17 is highly active in a wide range of acidic pH conditions (4.0‐7.0), similar to other β‐amylases, such as the major β‐amylases of barley.^6^

Figure 1C also shows the melting temperature of Tri a 17_active at different pH values, as measured by differential scanning fluorimetry. The protein is most stable in slightly acidic conditions. A maximum in melting temperature is seen at pH 5.5 with 59°C, with a tail off to lower values as pH increases and a sharp drop in stability at pH 4.

The IgE reactivity of the three forms of Tri a 17 was assessed in non‐denaturing RAST‐based IgE dot blot experiments with sera from 17 wheat food allergic patients. Tri a 17_clone and Tri a 17_inactive were recognized by 24% (4 of 17) of wheat food allergic patients with varying intensities, whereas the folded enzyme (Tri a 17_active) was recognized by 41% (7 of 17) of wheat food allergic patients, indicating the presence of conformational IgE epitopes as well as linear epitopes. Since the enzymatic activity depends on the correct fold, there is an apparent correlation between enzymatic and allergenic activity of the β‐amylase. IgE reactivity was specific to patients suffering from wheat food allergy. Non‐allergic individuals (patients 18 and 19), grass pollen allergic patients (patients 20 and 21), and baker's asthma patients (patients 22 and 23) did not exhibit IgE reactivity to either form of beta amylase (Figure 2A).

**Figure 2:**
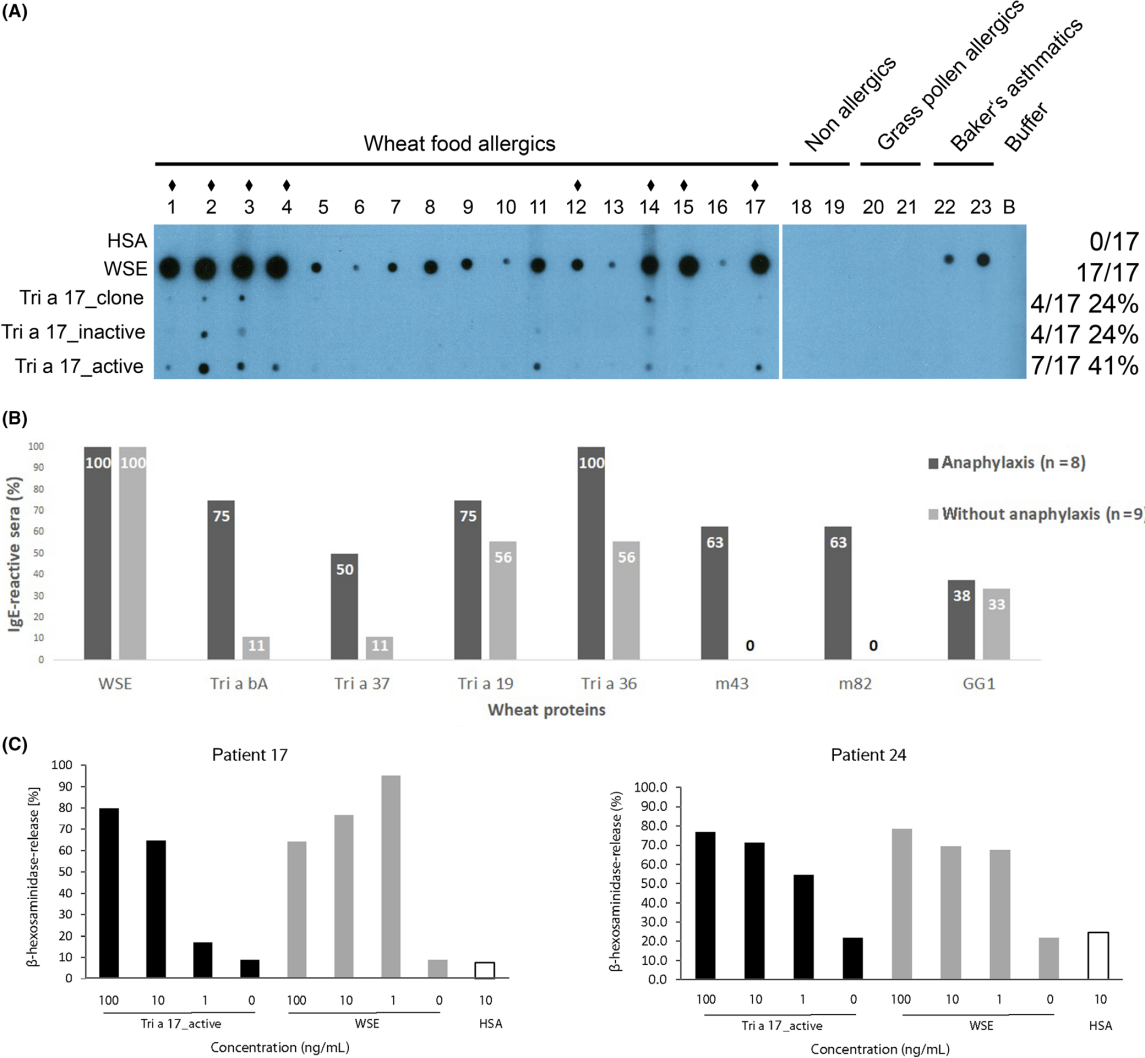
A, IgE reactivity of wheat β‐amylases (Tri a 17_clone, Tri a 17_inactive and Tri a 17_active). Nitrocellulose‐dotted human serum albumin (HSA), wheat seed extract (WSE), Tri a 17_clone and Tri a 17, in its inactive and active form, were tested with sera from wheat food allergic patients (1‐17). For control purposes, sera from non‐allergic individuals (18 and 19), grass pollen allergic patients (20 and 21) baker's asthma patients (22 and 23), or buffer alone (B) were included. Patients with a history of wheat‐induced anaphylaxis are indicated by ♦ above their number. B, IgE reactivity of wheat food allergic patients with and without anaphylaxis. IgE‐binding prevalences (*y*‐axis: percentage of IgE‐reactive sera) to wheat seed extract and wheat proteins (Tri a 17, Tri a 37, Tri a 19, Tri a 36, m43, m82, GG1) for patients with (black bars) and without anaphylaxis (gray bars). C, Allergenic activity of recombinant β‐amylase Tri a 17_active and wheat seed extract. RBL cells were loaded with serum IgE from Tri a 17_active‐reactive patients and incubated with increasing concentrations of Tri a 17_active (black) or WSE (gray) (100, 10, 1 ng/mL), buffer alone (0 ng/mL) or HSA (white) (10 ng/mL). β‐Hexosaminidase releases are displayed as percentages of total β‐hexosaminidase release on the *y*‐axes

Interestingly, 8 out of 17 wheat food allergic patients (47%) had a history of wheat‐induced anaphylaxis. Of those, six were among the seven β‐amylase positive patients. This correlation between anaphylaxis and Tri a 17 IgE recognition is statistically significant (*P* = 0.015). The relative risk of developing wheat‐induced anaphylaxis, estimated by logistic regression, indicates a 24‐fold higher probability for β‐amylase‐reactive patients (Methods, Online Repository). Figure 2B shows the IgE recognition frequencies for the patients with and without anaphylaxis for β‐amylase (Tri a 17) and other wheat allergens. High molecular weight glutenins (m43, m82,^7^ *P* = 0.009), low molecular weight glutenin (Tri a 36,^8^ *P* = 0.08), and alpha purothionin (Tri a 37,^9^ *P* = 0.13) also discriminate between patients with and without anaphylaxis, to varying degrees. However, omega 5 gliadin (Tri a 19, *P* = 0.62), gamma gliadin (GG1^10^), and wheat seed extract do not discriminate between patients with and without anaphylaxis.

Tri a 17_active was able to induce effector cell degranulation in basophil degranulation assays. Human FcεRI‐expressing RBL cells were passively sensitized with sera from wheat food allergic patients which showed IgE reactivity to Tri a 17 (Figure 2A, Figure S3). Subsequent incubation with increasing concentrations of Tri a 17_active or wheat seed extract (WSE) showed release for both wheat food allergic patients tested (Figure 2B).

Within a barley kernel, β‐amylases can reach 1%‐2% of the total protein in the starchy endosperm.^6^ Assuming similarly high expression in wheat, amylase concentrations that showed effector cell release (1 μg/L) are likely to be found in all flour‐containing foodstuffs.

The multiple sequence alignment of β‐amylase showed that highly homologous proteins occur in various plant species (Figure S2 in this article's Online Repository). Cross‐reactive antigens were detected in rye, maize, oat, spelt, barley, soy, sunflower, and rice using rabbit sera.

The protein was detected in all processed cereal products (ie, brown bread, rye bread, and rolls) including gluten‐free bread and even after baking, showing a remarkable persistence which may explain why it can induce severe reactions (Figure S4 in this article's Online Repository).

Based on our findings, wheat beta amylase has been given the official name “Tri a 17” by the WHO/IUIS allergen nomenclature subcommittee.

In summary, wheat β‐amylase can be classified as a class I food allergen that sensitizes via the gastrointestinal tract.

Wheat β‐amylase seems to be associated with severe allergic reactions upon wheat ingestion in sensitized patients and should be included in the panel of potential diagnostic marker molecules for severe wheat food allergy.

## CONFLICT OF INTEREST

Rudolf Valenta has received research grants from Biomay, Vienna, Austria and Viravaxx, Vienna, Austria and serves as a consultant for both companies. The other authors declare no conflicts of interest.
