# Supplementary material for: Three‐dimensional structure of the wheat β‐amylase Tri a 17, a clinically relevant food allergen
Source: Allergy. 2018 Dec 19;74(5):1009–13. doi: 10.1111/all.13696 (PMC6563530; doi:10.1111/all.13696)
Supplement: Supplementary file 1 [file ALL-74-1009-s001.zip › all13696-sup-0001-Supinfo.docx]

**Online repository**

**FIGURES**

**
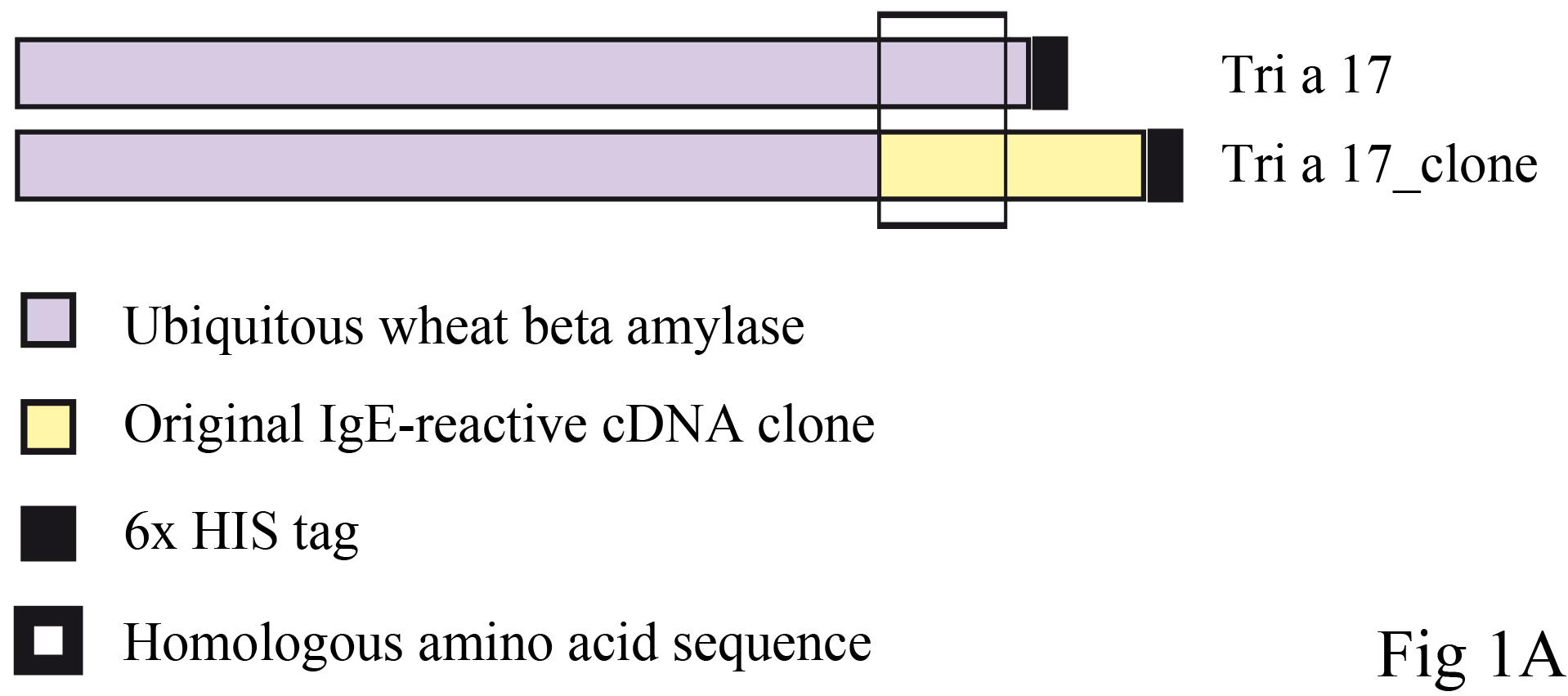
**

**Figure E1.** Illustration of two constructs for gene synthesis of wheat beta amylase Tri a 17 and Tri a 17_clone. Construct “Tri a 17” codes for wheat beta amylase CAA67128.1 (violet). In the construct “Tri a 17_clone” the C-terminal part has been replaced by the beta amylase-derived original IgE-reactive cDNA clone (yellow). The boxed part highlights well aligning amino acid sequences. Both constructs contain a hexahistidine-tag, visualized in black.


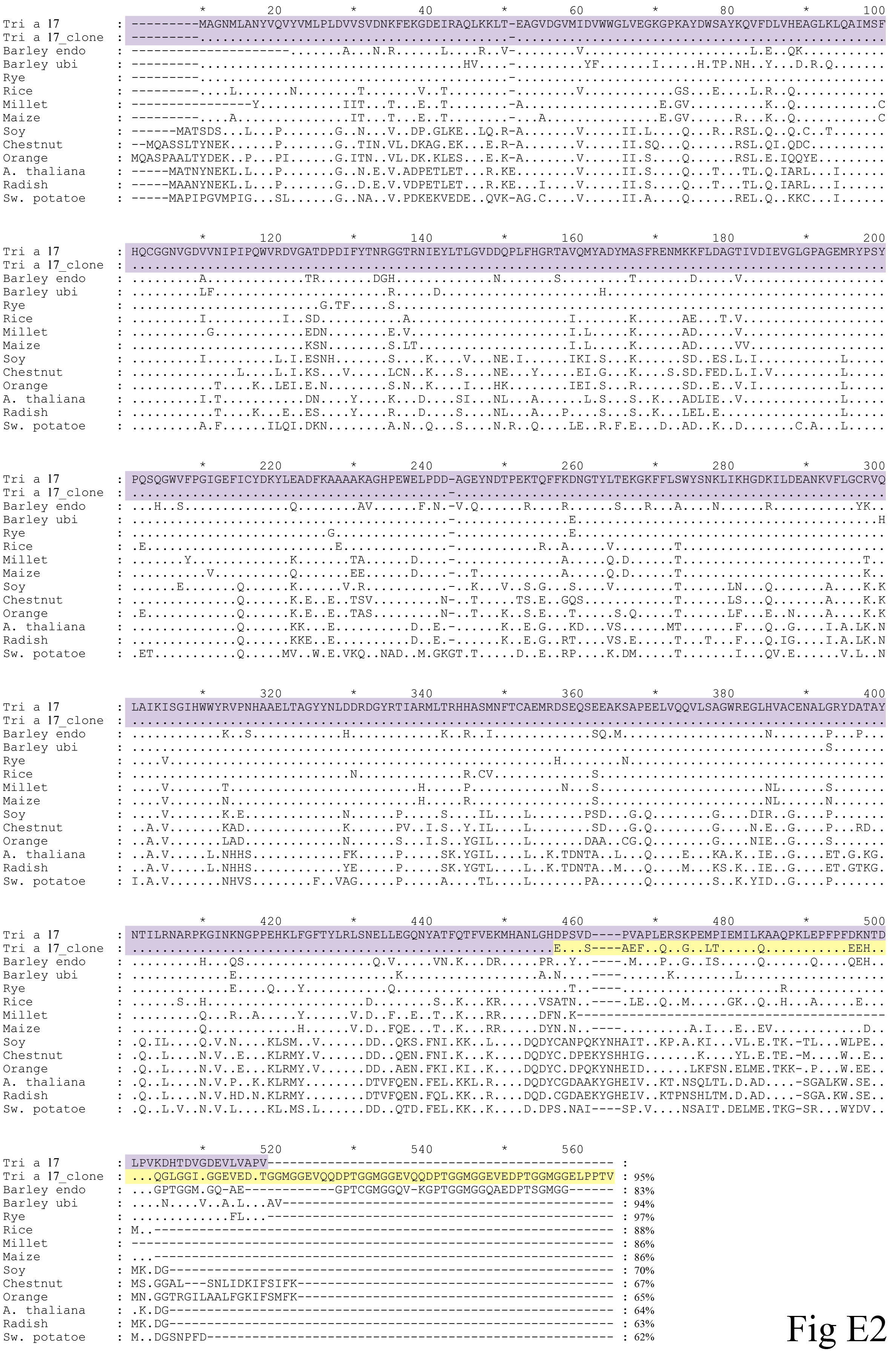


**Figure E2.** Sequence comparison of wheat beta amylase (*Triticum aestivum*, accession number CAA67128.1) with homologous proteins from other plants: rye (*Secale cereale,*. accession number CAA77817.1), barley endosperm-specific β-amylase (*Hordeum vulgare*, accession number AAO67356.1), barley ubiquitous β-amylase (*Hordeum vulgare*, accession number AAC64904.1), rice (*Oryza sativa*, accession number AAA33898.1), millet (*Sorghum bicolor*, accession number ADB81912.1), maize (*Zea mays*, accession number AAD15902.1), soy (*Glycine max*, accession number AAZ38831.1), chestnut (*Castanea crenata*, accession number AAK30294.1), orange (*Citrus trifoliata*, accession number AFQ33617.1), *Arabidopsis thaliana* ( accession number BAA07842.1), radish (*Raphanus sativus*, accession number BAH20736.1), sweet potatoe (*Ipomoea batatas*, accession number BAA02286.1). The sequences of the recombinant proteins, Tri a 17 and Tri a 17_clone, are highlighted again in violet and yellow (original IgE-reactive cDNA clone). The percentages of sequence identities to wheat beta amylase are shown at the right margins of the sequences.


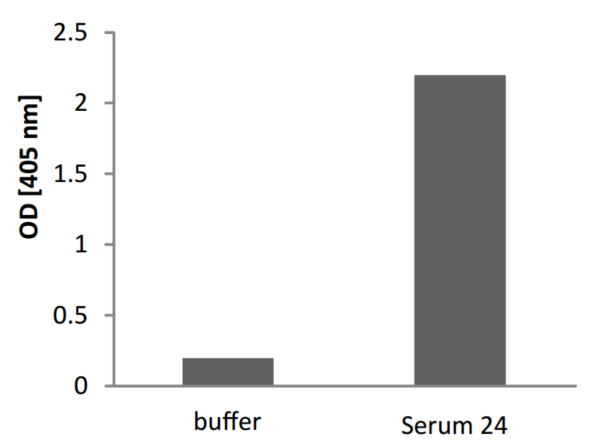


**Figure E3.** Specific IgE recognition of Tri a 17_active by patient #24. Serum from patient #24 and buffer without addition of serum was tested for IgE reactivity to Tri a 17_active by ELISA. OD values (y-axis) correspond to levels of bound IgE.


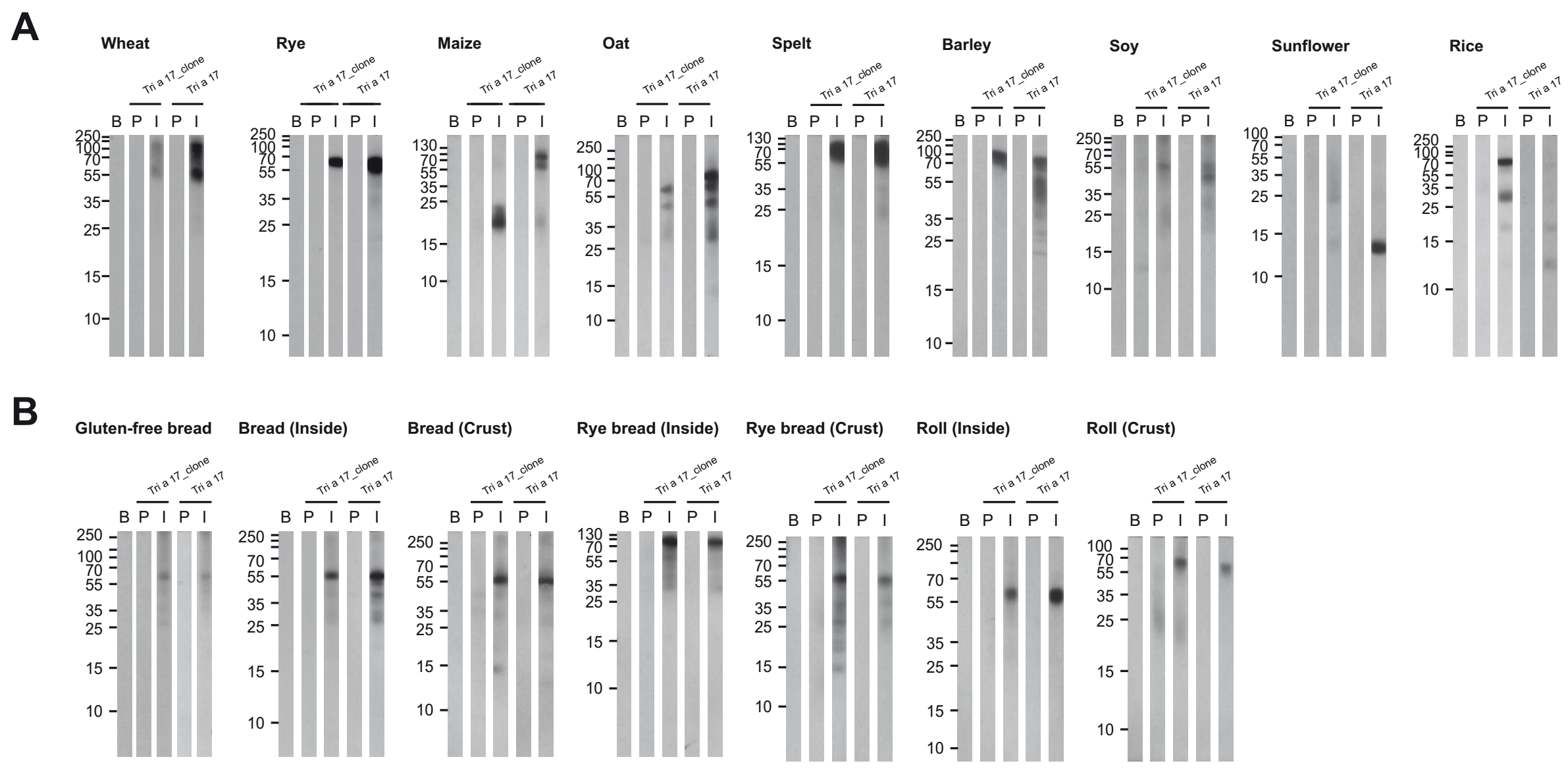


**Figure E4.** Detection of beta amylases (Tri a 17_clone, Tri a 17) in different cereals and types of bread. **(A)** Nitrocellulose-blotted extracts from cereals (wheat, rye, maize, oat, spelt, barley, soy, sunflower, rice) and **(B)** bread (gluten-free bread, inner part and crust of brown bread, rye bread and rolls) were incubated with Tri a 17_clone or Tri a 17 _inactive specific rabbit antibodies (lanes: I), the corresponding pre-immune serum (lanes: P) or buffer as control. Molecular weights are indicated on the left side in kilo Dalton (kDa).

**Table S1**

| Wavelength | 0.9717 |
| --- | --- |
| Resolution range | 26.96 - 2.0 (2.072 - 2.0) |
| Space group | P 4_3_ |
| Unit cell | 93.87 93.87 65.86 90 90 90 |
| Total reflections | 147815 (14537) |
| Unique reflections | 38550 (3721) |
| Multiplicity | 3.8 (3.8) |
| Completeness (%) | 91.32 (97.05) |
| Mean I/sigma(I) | 6.93 (3.76) |
| Wilson B-factor | 15 |
| R-merge | 0.1203 (0.2861) |
| R-meas | 0.1394 (0.3328) |
| R-pim | 0.06928 (0.1676) |
| CC1/2 | 0.986 (0.906) |
| CC* | 0.996 (0.975) |
| Reflections used in refinement | 35919 (3723) |
| Reflections used for R-free | 1253 (130) |
| R-work | 0.1582 (0.1735) |
| R-free | 0.1886 (0.1995) |
| CC(work) | 0.964 (0.924) |
| CC(free) | 0.958 (0.884) |
| Number of non-hydrogen atoms | 4474 |
| macromolecules | 3950 |
| solvent | 524 |
| Protein residues | 488 |
| RMS(bonds) | 0.003 |
| RMS(angles) | 0.59 |
| Ramachandran favored (%) | 98.35 |
| Ramachandran allowed (%) | 1.44 |
| Ramachandran outliers (%) | 0.21 |
| Rotamer outliers (%) | 1.68 |
| Clashscore | 1.54 |
| Average B-factor | 20.96 |
| macromolecules | 19.82 |
| solvent | 29.5 |

**Methods**

*Biological materials*

Wheat seeds were obtained from AGES (Österreichische Agentur für Gesundheit und 159 Ernährungssicherheit GmbH). Seeds from rye, maize, oat, spelt, barley, soy, sunflower and rice were bought at a local drugstore. Human serum albumin (HSA) was purchased from Behring (Marburg, Germany) and *E.coli* strain BL21 (DE3) from Stratagene (La Jolla, CA). Specific rabbit antibodies were raised by immunization of a rabbit each with purified recombinant beta amylase Tri a 17_inactive and Tri a 17_clone (200 μg per injection) using once Freund’s complete adjuvant and twice Freund’s incomplete adjuvant (Charles River, Kisslegg, Germany). Pre-immune sera were obtained from the rabbits before immunization.

*Preparation of protein extracts and immunoblots*

SDS-protein extracts for Western blotting (1) were prepared from equal amounts (weight) of seeds from wheat (cultivar Grandios), rye, maize, oat, spelt, barley, soy, sunflower and rice. Different sorts of bread (gluten-free bread, brown bread, rye bread, rolls) were bought at a local bakery and homogenized in a grinding machine (CTC, Clatronic International, Kempen, Germany). The powder obtained from 3 grams of tissue was extracted in 32 ml SDS-sample buffer by boiling for 10 minutes and centrifuged at 10 000 x g for 10 minutes at 4°C to obtain the cleared extracts. Aqueous wheat seed extract (WSE) was prepared as described.(2) The protein concentration was determined with the Micro BCA Protein Assay Kit (Pierce, Rockford, IL). Extracts were stored at ‑20°C until use.

Extracts were analyzed by SDS-PAGE with Coomassie-blue staining and blotted onto nitrocellulose. Nitrocellulose-blotted extracts were tested with beta amylase-specific rabbit antibodies, and for control purposes with the corresponding pre-immune serum (dilution 1:10.000) and buffer alone. Bound IgG antibodies were detected with ^125^I-labeled goat anti-rabbit antibodies (Perkin Elmer, Boston USA) and visualized by autoradiography.

*Patient’s sera*

Sera were obtained from 17 European wheat food allergic patients (Austria n=2, Finland n=7, Greece n=8). One patient (patient 24) from Japan was additionally included for rat basophil leukemia assays. Patients were diagnosed on the basis of a case history demonstrating that allergic symptoms (systemic anaphylaxis, airway symptoms, gastrointestinal symptoms, itching of the mouth, skin symptoms) were unambiguously related to the ingestion of wheat or wheat products.

A grading of symptoms and definition of anaphylaxis was performed according to the international position paper and recommendations for the definition of anaphylaxis. (3)(4) In 8 of the patients, open food challenge was performed and positive challenge results were obtained in 7 patients. For patients from Finland (patients #5-9 and #11), the PRACTall consensus criteria were used according to Sampson *et al* to describe clinical symptoms (3). Patient #5 had an immediate reaction upon open, oral wheat food challenge, showing symptoms in the skin (urticaria) and in the respiratory tract such as cough and rhinitis. Patient #6 reacted immediately with skin manifestations, such as urticaria and erythema. Patient #7 had a delayed type systemic allergic reaction, displaying lip edema, rhinitis, sneezing and itching of the mouth. Patient #8 demonstrated immediate symptoms, such as globus sensation and itching of the mouth. Patient #9 reacted immediately with gastrointestinal symptoms, such as abdominal pain and nausea. Patient #11 had delayed symptoms affecting the skin, showing itching and exacerbation of atopic eczema. Challenge tests in Greek patients were considered positive if objective signs were present (3). Patient #17 had sneezing, rhinitis and urticaria. Skin prick tests with wheat seed extract were performed in 14 patients, and were positive in all of them. For each patient IgE-mediated sensitization to wheat was confirmed by measurements of allergen-specific IgE (CAP-FEIA, Thermofisher, Uppsala, Sweden). Studies were performed on anonymized sera with permission of the Ethics Committee of the Vienna General Hospital.

*Expression and purification of recombinant wheat beta amylase in its active and inactive form*

The cDNA coding for the mature wheat beta amylase (accession number CAA67128.1, designated Tri a 17) as well as the beta amylase whose C-terminal part has been replaced by the beta amylase-derived original IgE-reactive cDNA clone (Tri a 17_clone) with an additional 3’ sequence coding for a hexahistidine tag were produced as synthetic genes with codons optimized for expression in *E. coli* and subcloned into the pET17b expression vector (GenScript, NJ, USA). The beta amylases, expressed in *Escherichia coli* BL21 (DE3) were purified by nickel affinity chromatography from the soluble fraction (Quiagen, Hilden, Germany) as previously described.(5) Recombinant allergens were stored in 10 mM NaH_2_PO_4_ buffer pH 7.0 at -20°C. The protein concentrations were determined by BCA assay (Pierce, Rockford, IL) and purity was assessed by Coomassie Blue-stained 14% SDS–PAGE. Proteins were analysed under reducing and non-reducing conditions.(6) By the use of this method, we obtained enzymatically inactive recombinant beta amylases.

In parallel, we expressed an active beta amylase using another protocol in *ΔtrxB, Δgor* strain *E.* *coli* (SHuffle, New England Biolabs) starting with an overnight culture in 2% glucose LB medium at 30°C. Main cultures were inoculated by adding overnight culture to a final OD_600_ of 0.1 into LB medium containing 0.5% glucose. Expression was induced by adding IPTG to a final concentration of 0.4 mM when the main cultures reached an OD_600_ of 0.6-0.8. Cultures were then either left at 30°C for 4 hours or cooled to 16°C and shaken overnight.

The *E. coli* pellets were sonicated for lysis in a 100 mM MES pH 6.5, 300 mM NaCl and 50mM imidazole buffer. The soluble fraction after centrifugation at 15 000g for 30 min was applied to an Immobilized Metal Affinity Chromatography (IMAC) column (HisTrap 5ml FF). After washing with 10 column volumes of lysis/washing buffer, the protein was eluted with the same buffer with increased imidazole concentration (300 mM).

For crystallization, the protein was further purified by bringing the solution to 40 % (v/v) ethanol, removing the precipitated proteins by centrifugation and changing the buffer of the beta amylase-containing supernatant to 50 mM MES pH 6, 50 mM NaCl using a micro centrifugation filter and stored at 4°C. In this case, proteins were analysed using 12% or 18% SDS-PAGE stained with Coomassie Blue.

For the activity and stability assays, a size exclusion chromatographic purification in 100mM NaCl, 10mM MES pH 6.5 was performed. (Superdex 200 10/300).

*Secondary structure determination*

Circular dichroism analyses were performed with protein concentrations of 0.5 mg/ml Tri a 17 in 25mM MES pH 6 and 25mM NaCl. Samples were analyzed in 0.2mm cuvettes using a Jasco J-715 spectropolarimeter at room temperature. The spectra were measured from 260 to 190nm, 0.2 nm data pitch, 50 nm per minute and a response time of 2 sec. Secondary structure predictions were performed using Dichroweb (7) and the CDSSTR method.

*Determination of enzymatic activity*

To achieve a range of conditions with different pH values but the same buffer system, a multicomponent buffer system was used [1 : 2 : 2 molar ratio of L-malic acid, MES and Tris; 1M total concentration]. Mixing of two buffers of the same buffer system, but with different pH values, one with pH 4 and one with pH 9, gives a reasonably linear pH gradient.(8)

The enzymatic activity of Tri a 17_active was measured by a modified version of the 3,5-dinitrosalicylic acid assay described by Fischer and Stein.(9) The modified protocol is as follows:

Substrate solution (45 μl, 1 % soluble starch [S9765, Sigma-Aldrich] in ddH2O) mixed with multicomponent buffer (8) (10 μl total) and protein solution (45 μl, 2 mg/l in 1 mM MES, 100 mM NaCl, pH 6.5) were equilibrated to 25 °C. The two solutions were mixed and shaken at 450 rpm. After 7 minutes, the reaction was quenched by adding stopping reagent (100 μl, 44 mM 3,5-dinitrosalicylic acid, 1.06 M sodium potassium tartrate, 0.4 M NaOH) and was subsequently placed at 100 °C for 5 minutes. Following, it was placed on ice for 3 minutes after which 1 ml of ddH_2_O was added. Blank samples were created by adding the stopping reagent before the protein solution.

The absorbance of the solution at 540 nm was measured in a 1 cm cell, using a Hitachi U-2001 spectrophotometer (Hitachi High-Technologies Corporation, Tokyo, Japan). The blank corrected absorbance was used to calculate the number of moles of maltose released (reducing ends equivalents) by means of a maltose standard curve, created with D(+)-maltose (Carl Roth, Karlsruhe, Germany). The activity of the enzyme in units was then calculated.

One unit of activity was defined as the release of one micromole of maltose per minute at 25°C, under the conditions outlined above.

*Thermostability*

The effect of pH on the melting temperature of Tri a 17_active was investigated using differential scanning fluorimetry. Protein solution (10 μl, 0.16 g/l in 10 mM MES, 100 mM NaCl), multicomponent buffer (8) (10 μl total) and SYPRO orange (5µl, 1:200 prediluted in ddH_2_O) were mixed in 96 well plates Using a C1000 Thermal Cycler (Bio Rad), the solution was heated 1°C per minute, from 25°C to 95°C. Fluorescence was measured every 0.5°C, using channel 2 of a CFX Real Time system (Bio Rad). The melting temperature T_m_ was calculated as the minimum of the first derivative of the fluorescence vs. the temperature.

*Crystallization and structure refinement*

For screening, the protein solution (in MES pH 6, 50 mM NaCl) was used in concentrations of 2-4 mg/ml. A mixture of 0.5 µl protein with 0.5 µl JCSG+ screen was performed in 96 well microbatch plates and covered with a combination of three parts paraffin to one part silicone oil.

The condition H3 (0.1M bis-Tris pH 5.5, 25% PEG 3350) was optimized and repeatable crystallization was achieved with 25% PEG 3350, 0.1M bis-Tris pH 5.0.

Molecular graphics and analyses were performed with PyMOl (The PyMOL Molecular Graphics System, Version 1.7.1 Schrödinger, LLC.).

The diffraction data were integrated using XDS (10), scaled and merged using SCALA of the CCP4 program suite (11). Molecular replacement was achieved using the PDB model 2XFR (12) of the barley amylase in the Phenix (Python-based Hierarchical ENvironment for Integrated Xtallography (13)) package, which was also used for refinement with alternating manual adaptation in *Coot* (14). The refined coordinates and structure factors have been deposited in the protein databank with the PDB-ID 6GER and experimental data are summarized in Table S1.

*Dot-blotting*

IgE-reactivity of wheat seed extract, beta amylases and additional wheat allergens was tested by non-denaturing, RAST-based IgE-dot blot experiments (5). Aliquots of each recombinant wheat allergens (0.5 μg/dot) were dotted onto nitrocellulose strips (Schleicher & Schuell, Dassel, Germany). Aliquots of HSA (0.5 μg/dot) and aqueous wheat seed extract (2 µg/dot) were dotted for control purposes. The strips were incubated with 1:10 diluted sera of 17 wheat food allergic patients, non-allergic individuals (n=2), grass pollen allergic patients (n=2), baker’s asthma patients (n=2) and a buffer control. Bound IgE antibodies were detected with ^125^I-labeled goat anti-human IgE antibodies (Demeditec Diagnostics, Kiel, Germany) and visualized by autoradiography (1). IgE-reactivity of omega-5-gliadin (f416: rTri a 19) was determined by ImmunoCAP measurements (ThermoScientific, Uppsala, Sweden) with a cut-off of 0.35 kUA/L.

*BAT assay*

Rat basophilic leukemia (RBL) cells expressing the α/β/γ subunits of the human FcεRI (15) were incubated with sera from patients with IgE reactivity to Tri a 17_active (patients 17 and 24 were chosen because they supplied sufficient serum for the experiment). Degranulation was induced by different concentrations of either Tri a 17_active, WSE or HSA as control. Released β-hexosaminidase was measured (16) and results are reported as percentage of total β-hexosaminidase release. All measurements were performed in triplicates.

*ELISA*

For certain patients, IgE reactivity to Tri a 17_active was tested by ELISA assay. Aliquots of 100 µl Tri a 17_active (5 µg/ml diluted in PBS) were coated over night at 4°C. After blocking for 3 hours with 3% BSA in PBST, serum (1:10 diluted) or buffer alone (negative control) was applied and incubated over night at 4°C. Bound IgE antibodies were detected as previously described.(17)

*Association of wheat allergens with anaphylaxis*

The odds ratio was calculated to determine the relative risk of a beta amylase positive patient to develop severe allergic symptoms. For this purpose, a cross tab was used to fulfill the current question. In this cross tab, eight patients had neither anaphylaxis nor IgE antibodies against beta amylase, whereas six patients have both. One patient had IgE but no anaphylaxis and two patients suffered from anaphylaxis but had no IgE antibodies. According to this result the relative risk was estimated by means of logistic regression as described (18).

The p-values were calculated by Fisher’s exact test in R (19) using the data found in figure 2B.

1. Valenta R, Duchene M, Ebner C, Valent P, Sillaber C, Deviller P et al. Profilins constitute a novel family of functional plant pan-allergens. *J Exp Med* 1992;**175**:377–385.

2. Baar A, Pahr S, Constantin C, Scheiblhofer S, Thalhamer J, Giavi S et al. Molecular and immunological characterization of Tri a 36, a low molecular weight glutenin, as a novel major wheat food allergen. *J Immunol* 2012;**189**:3018–3025.

3. Sampson HA, Gerth van Wijk R, Bindslev-Jensen C, Sicherer S, Teuber SS, Burks AW et al. Standardizing double-blind, placebo-controlled oral food challenges: American Academy of Allergy, Asthma &amp; Immunology–European Academy of Allergy and Clinical Immunology PRACTALL consensus report. *J Allergy Clin Immunol* 2012;**130**:1260–1274.

4. Sampson HA, Muñoz-Furlong A, Campbell RL, Adkinson NF, Allan Bock S, Branum A et al. Second symposium on the definition and management of anaphylaxis: Summary report - Second National Institute of Allergy and Infectious Disease/Food Allergy and Anaphylaxis Network Symposium. *Ann Emerg Med* 2006;**47**:373–380.

5. Pahr S, Constantin C, Mari A, Scheiblhofer S, Thalhamer J, Ebner C et al. Molecular characterization of wheat allergens specifically recognized by patients suffering from wheat-induced respiratory allergy. *Clin Exp Allergy* 2012;**42**:597–609.

6. Chen K-W, Blatt K, Thomas WR, Swoboda I, Valent P, Valenta R et al. Hypoallergenic Der p 1/Der p 2 combination vaccines for immunotherapy of house dust mite allergy. *J Allergy Clin Immunol* 2012;**130**:435–443.e4.

7. Whitmore L, Wallace BA. Protein secondary structure analyses from circular dichroism spectroscopy: Methods and reference databases. *Biopolymers* 2008;**89**:392–400.

8. Newman J. Novel buffer systems for macromolecular crystallization. *Acta Crystallogr Sect D Biol Crystallogr* 2004;**60**:610–612.

9. Fischer EH. *Biochemical Preperations*. 1961

10. Kabsch W. Xds. *Acta Crystallogr Sect D Biol Crystallogr* 2010;**66**:125–132.

11. Winn MD, Ballard CC, Cowtan KD, Dodson EJ, Emsley P, Evans PR et al. Overview of the CCP4 suite and current developments. *Acta Crystallogr Sect D Biol Crystallogr* 2011;**67**:235–242.

12. Rejzek M, Stevenson CE, Southard AM, Stanley D, Denyer K, Smith AM et al. Chemical genetics and cereal starch metabolism: structural basis of the non-covalent and covalent inhibition of barley β-amylase. *Mol Biosyst* 2011;**7**:718–730.

13. Adams PD, Afonine P V., Bunkóczi G, Chen VB, Davis IW, Echols N et al. PHENIX: A comprehensive Python-based system for macromolecular structure solution. *Acta Crystallogr Sect D Biol Crystallogr* 2010;**66**:213–221.

14. Emsley P, Lohkamp B, Scott WG, Cowtan K. Features and development of Coot. *Acta Crystallogr Sect D Biol Crystallogr* 2010;**66**:486–501.

15. Nakamura R, Uchida Y, Higuchi M, Nakamura R, Tsuge I, Urisu A et al. A convenient and sensitive allergy test: IgE crosslinking-induced luciferase expression in cultured mast cells. *Allergy* 2010;**65**:1266–1273.

16. Gieras A, Focke-Tejkl M, Ball T, Verdino P, Hartl A, Thalhamer J et al. Molecular determinants of allergen-induced effector cell degranulation. *J Allergy Clin Immunol* 2007;**119**:384–390.

17. Resch Y, Weghofer M, Seiberler S, Horak F, Scheiblhofer S, Linhart B et al. Molecular characterization of Der p 10: a diagnostic marker for broad sensitization in house dust mite allergy. *Clin Exp Allergy* 2011;**41**:1468–1477.

18. Pahr S, Constantin C, Papadopoulos NG, Giavi S, Mäkelä M, Pelkonen A et al. α-Purothionin, a new wheat allergen associated with severe allergy. *J Allergy Clin Immunol* 2013;**132**:1000–1003.e4.

19. R Development Core Team. R: A language and environment for statistical computing. 2015. doi:10.1007/978-3-540-74686-7
